# Validation of automated image-guided programming in STN-DBS for Parkinson's disease

**DOI:** 10.3389/fnhum.2026.1845500

**Published:** 2026-05-20

**Authors:** Atsushi Umemura, Hideki Mizuno, Mina Maki, Yasuaki Echizen, Atsuo Masago, Akihide Kondo

**Affiliations:** 1 Department of Neurosurgery, Juntendo University, Tokyo, Japan; 2 Department of Neurosurgery, Ookuma Hospital, Nagoya, Japan; 3 Department of Neurology, Ookuma Hospital, Nagoya, Japan

**Keywords:** current steering, deep brain stimulation, image-guided programming, Parkinson's disease, subthalamic nucleus

## Abstract

**Background:**

Current steering with multiple independent current control (MICC) and directional leads has expanded programming options in subthalamic nucleus deep brain stimulation (STN-DBS), but has also increased programming complexity. Manual image-guided programming (mIGP) using patient-specific anatomical information facilitates optimization of stimulation settings. More recently, algorithm-based platforms for automated image-guided programming (aIGP) have been developed, although their clinical validity remains unclear.

**Methods:**

Thirteen patients with Parkinson's disease who had undergone STN-DBS using a MICC directional lead system and had stable motor symptoms under mIGP established with StimviewTM XT were enrolled. aIGP was performed using IlluminaTM 3D under two conditions. In Part 1, the target region was the STN and the avoid region was the internal capsule. In Part 2, the target region was the STN, whereas the red nucleus and substantia nigra were designated as avoid regions to direct stimulation toward the dorsolateral STN. Stimulation amplitude, pulse width, and frequency were unchanged; only the spatial distribution of stimulation was modified. Motor symptoms were assessed using the Unified Parkinson's Disease Rating Scale part III (UPDRS III) immediately before and 1 hour after switching from mIGP to aIGP-derived settings.

**Results:**

In Part 1, 4 of 13 patients showed obvious worsening shortly after switching and were immediately returned to mIGP. Of the remaining 9, 8 reverted to mIGP within 1 week, whereas 1 continued aIGP because of improved speech difficulty. Mean UPDRS III scores changed from 16.4 ± 1.7 to 19.6 ± 2.5 (*P* = 0.16). In Part 2, 3 of 13 patients developed tremor shortly after switching and were immediately returned to mIGP. The remaining 10 all reverted to mIGP within 2 weeks. Mean UPDRS III scores changed from 15.5 ± 1.5 to 18.3 ± 1.8 (*P* = 0.07).

**Conclusion:**

Although aIGP rapidly generated anatomically plausible current-steering configurations, most patients did not maintain these settings, indicating limited clinical acceptability compared with expert-guided mIGP. Further refinement, particularly with more selective emphasis on the dorsolateral STN, will be necessary before aIGP can be considered a reliable alternative.

## 1 Introduction

Deep brain stimulation of the subthalamic nucleus (STN-DBS) is an established surgical treatment for patients with Parkinson's disease (PD) whose motor symptoms are inadequately controlled with medical therapy. Its clinical efficacy depends not only on appropriate patient selection and accurate lead placement, but also on optimal postoperative programming. In particular, the advent of multiple independent current control (MICC) technology in combination with directional leads has expanded the capability for current steering, enabling the stimulation field to be shaped more precisely according to individual STN anatomy. This technological advancement has the potential to improve motor outcomes while reducing stimulation-induced adverse effects (Dembek et al., 2017; Merola et al., 2020; Schnitzler et al., 2022; Umemura et al., 2023; Maçaneiro et al., 2024).

Accumulating anatomical, electrophysiological, and clinical evidence suggests that the most effective stimulation site for alleviating parkinsonian motor symptoms lies within the dorsolateral portion of the STN or its adjacent lateral region (Benarroch, 2008; Guo et al., 2013; Zhang et al., 2021; Akram et al., 2017; Garcia-Garcia et al., 2016; Vitek et al., 2022). Accordingly, programming strategies that selectively direct stimulation toward the dorsolateral STN are considered highly relevant for maximizing therapeutic benefit. However, the growing flexibility of current steering has also made programming increasingly complex. Conventional symptom-guided programming, which relies on repeated clinical testing of multiple contact configurations, is often labor-intensive, time-consuming, and highly dependent on the experience of the clinician.

To address these challenges, image-guided programming (IGP) has been introduced as an anatomy-based approach for optimizing DBS settings. By integrating preoperative magnetic resonance imaging with postoperative lead localization, IGP allows visualization of the spatial relationship between the implanted lead and patient-specific anatomical structures, including the STN and surrounding nuclei. Previous studies have shown that IGP can facilitate more efficient programming and can achieve clinical outcomes comparable or superior to those obtained with conventional programming (Pavese et al., 2020; Waldthaler et al., 2021; Lange et al., 2021; Vilkhu et al., 2023; Rolland et al., 2024; Torres et al., 2024; Aldred et al., 2025). In particular, recent work using Stimview^TM^ XT (Boston Scientific, Valencia, CA, USA) demonstrated that manual IGP (mIGP) can refine current steering toward the dorsolateral STN and improve motor outcomes even in patients whose symptoms had already stabilized under conventional programming (Umemura et al., 2025).

More recently, algorithm-based software platforms have been developed to automate the IGP process (Malekmohammadi et al., 2022; Maghzi et al., 2025). In principle, automated IGP (aIGP) may further reduce programming burden by generating stimulation settings objectively and rapidly on the basis of predefined target and avoidance regions. Such an approach could improve workflow efficiency and reduce dependence on individual programmer expertise. Nevertheless, whether aIGP can produce stimulation settings that are clinically comparable to those derived from expert-guided mIGP remains unclear. In particular, it is unknown whether automated algorithms can adequately capture the practical programming principle of selectively steering stimulation toward the dorsolateral STN while avoiding spread to adjacent structures.

In the present study, we evaluated the short-term clinical validity and acceptability of aIGP using Illumina^TM^ 3D (Boston Scientific, Valencia, CA, USA) in patients with Parkinson's disease who had undergone STN-DBS with directional leads and had stable motor symptoms under mIGP settings generated with Stimview^TM^ XT. By comparing the clinical tolerability and short-term acceptability of aIGP-derived settings with previously established mIGP settings, we aimed to determine the current clinical utility and limitations of this newly introduced algorithm-based programming approach. Our intention was not to test IGP as a concept, but rather to assess whether the current aIGP could achieve outcomes comparable to those of expert-refined mIGP.

## 2 Method

### 2.1 Participants

This study enrolled 13 patients with advanced PD who underwent bilateral STN-DBS using a MICC directional lead system (Boston Scientific, Valencia, CA, USA) for levodopa-induced motor complications and had been followed for more than 6 months after surgery. The cohort comprised nine men and four women, with a mean age of 63.8 ± 2.1 years (mean ± standard deviation [SD]). In all patients, stimulation settings had previously been optimized by mIGP using Stimview^TM^ XT, and motor symptoms were clinically stable at study entry without evident diurnal fluctuation or dyskinesia.

All participants provided written informed consent. The study was approved by the Institutional Review Board of our institution (approval no. 25-03).

### 2.2 Surgical procedure

The surgical procedure has been described in detail previously. In brief, bilateral directional leads were implanted into the STN using stereotactic techniques based on preoperative imaging and intraoperative physiological guidance, as reported in our earlier study (Umemura et al., 2013).

### 2.3 Manual IGP with Stimview^*TM*^ XT

Manual IGP with Stimview^TM^ XT was performed as previously described (Umemura et al., 2025). Briefly, preoperative magnetic resonance imaging and postoperative computed tomography were integrated to visualize the patient-specific spatial relationship between the STN, surrounding structures, and the implanted leads. Based on these reconstructed anatomical data, current steering was manually adjusted using both horizontal and vertical steering to shape the stimulation field so that it preferentially covered the dorsolateral STN while minimizing current spread beyond the nucleus. As in our previous report, stimulation amplitude, pulse width, and frequency were, in principle, not altered during this optimization process.

### 2.4 Automated IGP with Illumina^*TM*^ 3D

Automated IGP was performed using Illumina^TM^ 3D. Because this software requires predefined target and avoid regions, two programming conditions were evaluated.

In Part 1, the target region was defined as the STN and the avoid region as the internal capsule, with the intention of reducing stimulation-induced pyramidal tract adverse effects.

In Part 2, the target region was again defined as the STN, whereas the red nucleus, located medial to the STN, and the substantia nigra, located inferior to the STN, were designated as avoid regions in an attempt to direct the stimulation field toward the dorsolateral STN. The two parts were conducted on separate outpatient occasions at least 2 months apart.

In both parts, stimulation amplitude, pulse width, and frequency were kept identical to those of the established mIGP settings, and only the spatial distribution of stimulation was modified. Thus, the present study was designed to assess the clinical effect of changing the stimulation field alone.

### 2.5 Clinical assessment and follow-up

Motor symptoms were evaluated using the Unified Parkinson's Disease Rating Scale part III (UPDRS III) immediately before application of the aIGP-derived settings and again 1 h after reprogramming, in accordance with the assessment framework used in our previous study of IGP. If a participant reported obvious worsening of symptoms after switching to the aIGP-derived settings, the UPDRS III assessment was completed and the stimulation settings were then immediately returned to the original mIGP program.

If the participant reported no subjective worsening or noted improvement after application of aIGP, the patient was allowed to return home with access to both the aIGP- and mIGP-derived programs via the patient controller and was instructed to switch freely between the two settings according to symptom status. Participants were scheduled to return to the outpatient clinic 1 month later. If the aIGP-derived settings had been continued until that visit, UPDRS III was to be reassessed at that time.

### 2.6 Statistical analysis

Changes in UPDRS III motor scores before and after application of aIGP were analyzed using a two-tailed paired *t*-test. A two-sided *P*-value of < 0.05 was considered statistically significant.

## 3 Results

### 3.1 Stimulation settings in manual and automated IGP

**Figure 1:**
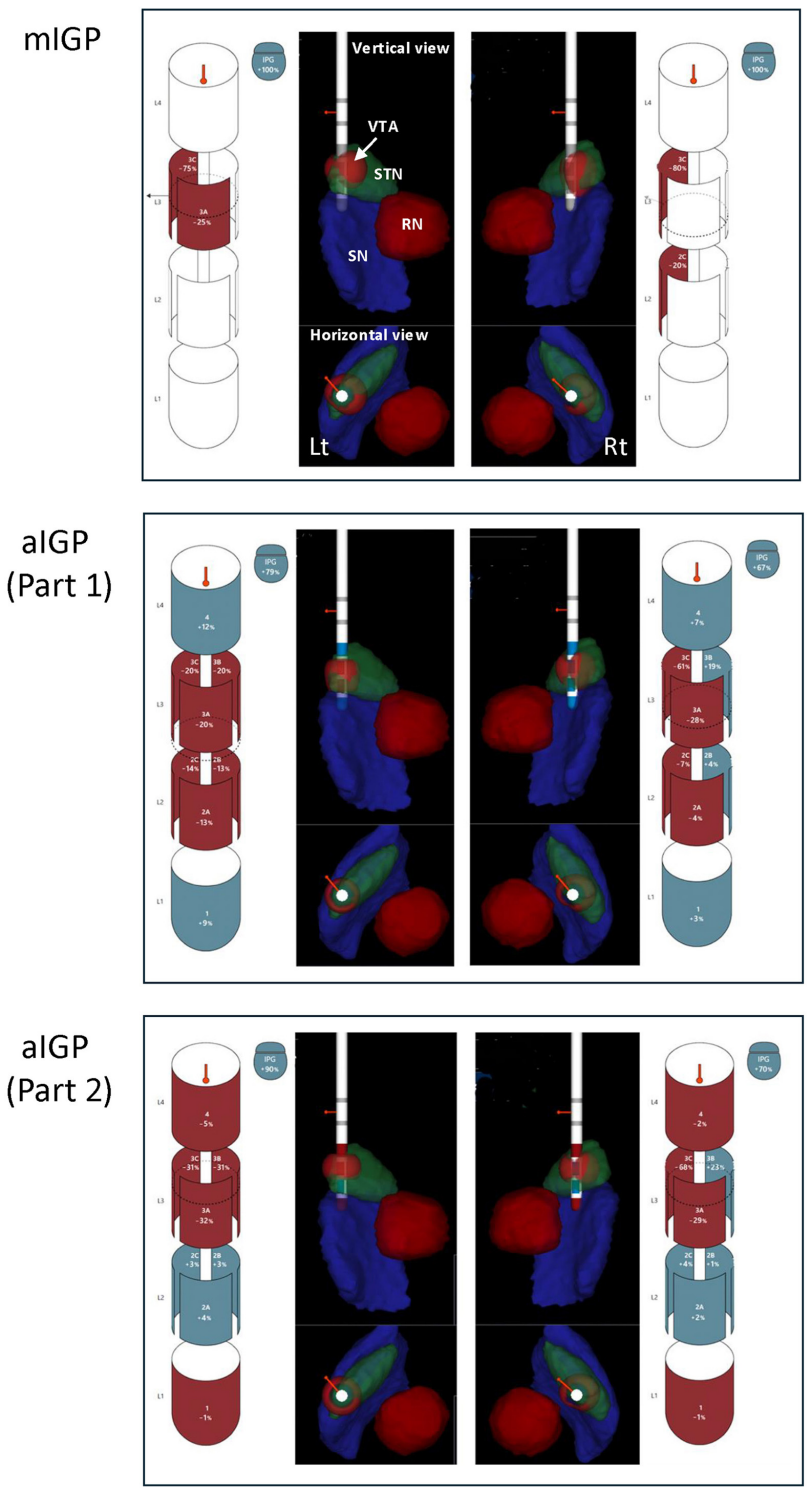
Manual and automated image-guided programming in Case 1. This 71-year-old man, evaluated 70 months after DBS surgery, had shown a favorable clinical course without evident motor fluctuations or dyskinesia under the stimulation settings established by mIGP using Stimview XT (upper panel). During Part 1 of the present clinical study, after switching to aIGP (middle panel), he was observed in the outpatient waiting area and gradually began to complain of malaise, followed by generalized rigidity and inability to move. His UPDRS part III motor score worsened from 20 to 42 points. His symptoms promptly improved after the stimulation settings were immediately returned to the original mIGP program. Comparison of the stimulation fields between mIGP and aIGP showed that, on the left side, the field that had been focused on the dorsolateral STN shifted ventrally, whereas on the right side, the field that had been directed laterally within the STN became oriented somewhat more anteriorly. In Part 2, which was conducted on a separate occasion, the patient reported a mild sense of constriction after switching to aIGP (lower panel). His UPDRS part III motor score worsened from 20 to 25 points; however, because this change was considered tolerable, he was allowed to return home and was followed clinically. After returning home, he again felt unwell and switched back to the original mIGP settings using the patient controller. In Part 2, the ventral shift of the stimulation field on the left side observed in Part 1 was no longer present, whereas on the right side the stimulation field shifted dorsally and was directed somewhat more anteriorly. STN, subthalamic nucleus; RN, red nucleus; SN, substantia nigra; VTA, volume of tissue activated.

The stimulation settings generated by mIGP and by aIGP in Part 1 and Part 2 for all 13 patients are summarized in Table 1. A representative case is shown in Figure 1. Under mIGP, all active contacts were configured as cathodes, with the implantable pulse generator (IPG) serving as the 100% anode, thereby creating a monopolar stimulation setting. In contrast, under aIGP, cathodic and/or anodic assignments were distributed across nearly all lead contacts. Although the IPG remained the principal anode in most instances, a portion of the anodic current was also assigned to lead contacts, resulting in a more spatially focused semi-monopolar configuration. These aIGP-derived contact configurations were generated automatically by the algorithm and appeared to differ substantially from programming patterns that would ordinarily be selected in routine clinician-guided DBS programming.

**Table 1:** Stimulation settings by mIGP and aIGP.

No.	Age/sex	Manual image-guided program by Stimview XT	Automated image-guided program by Illumina 3D Part 1 (Target; STN, Avoid; Capsula interna)	Automated image-guided program by Illumina 3D Part 2 (Target; STN, Avoid; Red nucleus and Substantia Nigra)
1	71/M	Lt: 3A (−25%) 3C (−75%) IPG (+100%), 2.2 mA, 60 μs, 130 Hz Rt: 2C (−20%) 3C (−80%) IPG (+100%), 3.8 mA, 60 μs, 130 Hz	Lt: 1 (+11%) 2A (−17%) 2B (−17%) 2C (−17%) 3A (−17%) 3B (−16%) 3C (−16%) 4 (+10%) IPG (+79%), 2.2 mA, 60 μs, 130 Hz Rt: 1 (+4%) 2A (−9%) 2B (+5%) 2C (−9%) 3A (−40%) 3B (+19%) 3C (−42%) 4 (+8%) IPG (+64%), 3.8 mA, 60 μs, 130 Hz	Lt: 1 (−1%) 2A (+4%) 2B (+3%) 2C (+3%) 3A (−32%) 3B (−31%) 3C (−31%) 4 (−5%) IPG (+90%), 2.2 mA, 60 μs, 130 Hz Rt: 1 (−1%) 2A (+2%) 2B (+1%) 2C (+4%) 3A (−29%) 3B (+23%) 3C (−68%) 4 (−2%) IPG (+70%), 3.8 mA, 60 μs, 130 Hz
2	66/M	Lt: 2A (−8%) 2B (−6%) 2C (−6%) 3A (−28%) 3B (−26%) 3C (−26%) IPG (+100%), 2.9 mA, 60 μs, 130 Hz Rt: 3B (−25%) 3C (−75%) IPG (+100%), 2.5 mA, 60 μs, 130 Hz	Lt: 1 (+11%) 2A (−28%) 2B (−28%) 2C (−28%) 3A (−5%) 3B (−6%) 3C (−5%) 4 (+5%) IPG (+84%), 2.9 mA, 60 μs, 130 Hz Rt: 1 (+7%) 2A (+13%) 2B (−81%) 2C (−11%) 3A (+2%) 3B (−7%) 3C (−1%) 4 (+3%) IPG (+75%), 2.5 mA, 60 μs, 130 Hz	Lt: 1 (+4%) 2A (−3%) 2B (−3%) 2C (−4%) 3A (−30%) 3B (−30%) 3C (−30%) 4 (+10%) IPG (+86%), 2.9 mA, 60 μs, 130 Hz Rt: 1 (−2%) 2A (−1%) 2B (+12%) 3A (+14%) 3B (−56%) 3C (+5%) 4 (−41%) IPG (+69%), 2.5 mA, 60 μs, 130 Hz
3	69/F	Lt: 3A (−50%) 3B (−50%) IPG (+100%), 3.1 mA, 60 μs, 130 Hz Rt: 2B (−8%) 2C (−22%) 3B (−18%) 3C (−52%) IPG (+100%), 3.0 mA, 60 μs, 130 Hz	Lt: 1 (+6%) 2A (−83%) 2B (−12%) 2C (+15%) 3A (−4%) 3B (−1%) 3C (+2%) 4 (+2%) IPG (+75%), 3.1 mA, 60 μs, 130 Hz Rt: 1 (−2%) 2A (−33%) 2B (−32%) 2C (−33%) 3A (+3%) 3B (+2%) 3C (+3%) IPG (+92%), 3.0 mA, 60 μs, 130 Hz	Lt: 1 (−1%) 2A (+7%) 2B (+2%) 3A (−75%) 3B (−20%) 3C (+20%) 4 (−4%) IPG (+71%), 3.1 mA, 60 μs, 130 Hz Rt: 1 (+3%) 2A (−9%) 2B (^*^5%) 2C (−7%) 3A (−48%) 3B (+19%) 3C (−36%) 4 (+8%) IPG (+65%), 3.0 mA, 60 μs, 130 Hz
4	70/F	Lt: 2B (−50%) 2C (−50%) IPG (+100%), 3.3 mA, 60 μs, 130 Hz Rt: 2B (−25%) 2C (−25%) 3B (−25%) 3C (−25%) IPG (+100%), 3.7 mA, 60 μs, 130 Hz	Lt: 1 (+7%) 2A (+20%) 2B (−39%) 2C (−48%) 3A (+5) 3B (−6%) 3C (−7%) IPG (+65%), 3.3 mA, 60 μs, 130 Hz Rt: 1 (−1%) 2A (+1%) 2B (+5%) 2C (+1%) 3A (+18%) 3B (−82%) 3C (−15%) 4 (−2%) IPG (+75%), 3.7 mA, 60 μs, 130 Hz	Lt: 1 (−5%) 2A (+25%) 2B (−46%) 2C (−49%) 3B (+3%) 3C (+4%) IPG (+68%), 3.3 mA, 60 μs, 130 Hz Rt: 1 (−2%) 2A (+14%) 2B (−91%) 2C (−7%) 3A (+1%) 3B (+6%) IPG (+92%), 3.7 mA, 60 μs, 130 Hz
5	58/M	Lt: 3B (−25%) 3C (−75%) IPG (+100%), 2.9 mA, 60 μs, 179 Hz Rt: 3A (−25%) 3C (−75%) IPG (+100%), 3.7 mA, 60 μs, 179 Hz	Lt: 1 (+5%) 2A (+20%) 2B (−39%) 2C (−48%) 3A (+5%) 3B (−6%) 3C (−7%) 4 (+11%) IPG (+84%), 2.9 mA, 60 μs, 179 Hz Rt: 2A (+6%) 2C (+1%) 3A (−87%) 3B (+16%) 3C (−11%) 4 (−2%) IPG (+77%), 3.7 mA, 60 μs, 179 Hz	Lt: 2A (+2%) 2B (+1%) 2C (+2%) 3A (−34%) 3B (−33%) 3C (−33%) 4 (+2%) IPG (+93%), 2.9 mA, 60 μs, 179 Hz Rt: 1 (−2%) 2A (+10%) 2C (+2%) 3A (−75%) 3B (+18%) 3C (−11%) 4 (−12%) IPG (+70%), 3.7 mA, 60 μs, 179 Hz
6	65/M	Lt: 3A (−20%) 3B (−20%), 3C (−20%) 4 (−40%) IPG (+100%), 2.7 mA, 60 μs, 130 Hz Rt: 3A (−45%), 3C (−15%) 4 (−40%) IPG (+100%), 3.6 mA, 60 μs, 130 Hz	Lt: 1 (−2%) 2A (+4%) 2B (+9%) 2C (−2%) 3A (−21%) 3B (−49%) 3C (+23%) 4 (−26%) IPG (+64%), 2.7 mA, 60 μs, 130 Hz Rt: 1 (−2%) 2A (+12%) 2C (+1%) 3A (−84%) 3B (+8%) 3C (+4%) 4 (−14%) IPG (+75%), 3.6 mA, 60 μs, 130 Hz	Lt: 1 (−1%) 2A (+2%) 2B (+2%) 2C (+2%) 3A (−5%) 3B (−5%) 3C (−5%) 4 (−84%) IPG (+94%), 2.7 mA, 60 μs, 130 Hz Rt: 1 (−2%) 2A (+11%) 2B (−1%) 2C (+2%) 3A (−62%) 3B (+20%) 3C (−11%) 4 (−24%) IPG (+67%), 3.6 mA, 60 μs, 130 Hz
7	71/M	Lt: 2A (−75%) 2B (−25%) IPG (+100%), 3.5 mA, 60 μs, 130 Hz Rt: 2A (−75%) 2B (−25%) IPG (+100%), 3.9 mA, 60 μs, 130 Hz	Lt: 1 (−4%) 2A (−32%) 2B (−32%) 2C (−32%) 3A (+3%) 3B (+3%) 3C (+3%) IPG (+91%), 3.5 mA, 60 μs, 130 Hz Rt: 1 (+3%) 2A (−100%) 2B (+4%) 2C (+4%) 3A (+1%) 3B (+1%) 4 (+1%) IPG (+86%), 3.9 mA, 60 μs, 130 Hz	Lt: 1 (+3%) 2A (−10%) 2B (+3%) 2C (−2%) 3A (−74%) 3B (+14%) 3C (−14%) 4 (+8%) IPG (+72%), 3.5 mA, 60 μs, 130 Hz Rt: 1 (+11%) 2A (−63%) 2B (+2%) 2C (+1%) 3A (−37%) 3B (+1%) 4 (+9%) IPG (+76%), 3.9 mA, 60 μs, 130 Hz
8	52/F	Lt: 3C (−100%) IPG (+100%), 2.5 mA, 60 μs, 130 Hz Rt: 3B (−100%) IPG (+100%), 2.8 mA, 60 μs, 130 Hz	Lt: 1 (+4%) 2A (+1%) 2C (−11%) 3A (+3%) 3B (+2%) 3C (−89%) 4 (+9%) IPG (+81%), 2.5 mA, 60 μs, 130 Hz Rt: 1 (+8%) 2A (+12%) 2B (−25%) 2C (−25%) 3A (+11%) 3B (−25%) 3C (−25%) 4 (+8%) IPG (+61%), 2.8 mA, 60 μs, 130 Hz	Lt: 1 (−2%) 2A (+1%) 2C (+9%) 3A (−14%) 3B (+19%) 3C (−75%) 4 (−9%) IPG (+71%), 2.5 mA, 60 μs, 130 Hz Rt: 1 (+1%) 2A (+3%) 2B (−1%) 2C (−1%) 3A (+23%) 3B (−49%) 3C (−49%) 4 (+4%) IPG (+69%), 2.8 mA, 60 μs, 130 Hz
9	70/M	Lt: 2A (−15%) 2C (−45%) 3A (−10%) 3C (−30%) IPG (+100%), 1.6 mA, 60 μs, 130 Hz Rt: 2 (−20%) 3 (−80%) IPG (+100%), 3.5 mA, 60 μs, 130 Hz	Lt: 1 (+11%) 2A (−27%) 2B (−27%) 2C (−28%) 3A (−6%) 3B (−6%) 3C (−6%) 4 (+5%) IPG (+84%), 1.6 mA, 60 μs, 130 Hz Rt: 1 (+6%) 2A (−49%) 2B (−42%) 2C (+21%) 3A (−5%) 3B (−4%) 3C (+4%) 4 (+2%) IPG (+67%), 3.5 mA, 60 μs, 130 Hz	Lt: 2A (+3%) 2B (+2%) 2C (+2%) 3A (−33%) 3B (−33%) 3C (−33%) 4 (−1%) IPG (+93%), 1.6 mA, 60 μs, 130 Hz Rt: 1 (−2%) 2A (+3%) 2B (+10%) 2C (−1%) 3A (−15%) 3B (−64%) 3C (+20%) 4 (−18%) IPG (+67%), 3.5 mA, 60 μs, 130 Hz
10	66/F	Lt: 3A (−90%) 4 (−10%) IPG (+100%), 3.8 mA, 60 μs, 130 Hz Rt: 3A (−100%) IPG (+100%), 2.4 mA, 60 μs, 130 Hz	Lt: 1 (+6%) 2A (−13%) 2B (−20%) 2C (+8%) 3A (−26%) 3B (−41%) 3C (+15%) 4 (+9%) IPG (+62%), 3.8 mA, 60 μs, 130 Hz Rt: 1 (+1%) 2B (−1%) 2C (+2%) 3A (−11%) 3B (−88%) 3C (+15%) 4 (+4%) IPG (+78%), 2.4 mA, 60 μs, 130 Hz	Lt: 1 (+1%) 2A (−2%) 2B (−2%) 2C (+3%) 3A (−42%) 3B (−54%) 3C (+23%) 4 (+4%) IPG (+69%), 3.8 mA, 60 μs, 130 Hz Rt: 1 (−1%) 2A (+2%) 2B (+9%) 2C (−2%) 3A (−8%) 3B (−46%) 3C (+22%) 4 (−43%) IPG (+67%), 2.4 mA, 60 μs, 130 Hz
11	56/M	Lt: 3A (−75%) 3C (−25%) IPG (+100%), 2.8 mA, 60 μs, 130 Hz Rt: 2A (−38%) 2C (−12%) 3A (−38%) 3C (−12%) IPG (+100%), 3.6 mA, 60 μs, 130 Hz	Lt: 1 (+2%) 2A (−5%) 2B (−2%) 2C (+3%) 3A (−76%) 3B (−17%) 3C (+16%) 4 (+6%) IPG (+73%), 2.8 mA, 60 μs, 130 Hz Rt: 1 (+8%) 2A (−34%) 2B (+1%) 2C (+1%) 3A (−66%) 3B (+1%) 3C (+1%) 4 (+12%) IPG (+76%), 3.6 mA, 60 μs, 130 Hz	Lt: 1 (−2%) 2A (+9%) 2B (+2%) 3A (−74%) 3B (−16%) 3C (+19%) 4 (−8%) IPG (+70%), 2.8 mA, 60 μs, 130 Hz Rt: 1 (+1%) 2A (+1%) 2B (+1%) 3A (−100%) 3B (+8%) 4 (+4%) IPG (+85%), 3.6 mA, 60 μs, 130 Hz
12	49/M	Lt: 2A (−22%) 2B (−8%) 3A (−52%) 3B (−18%) IPG (+100%), 4.1 mA, 50 μs, 130 Hz Rt: 2A (−50%) 3A (−50%) IPG (+100%), 2.8 mA, 50 μs, 130 Hz	Lt: 1 (−1%) 2A (+8%) 2B (+1%) 3A (−84%) 3B (−10%) 3C (+16%) 4 (−5%) IPG (+75%), 4.1 mA, 50 μs, 130 Hz Rt: 1 (+10%) 2A (−49%) 2B (−3%) 2C (+4%) 3A (−45%) 3B (−3%) 3C (+4%) 4 (+10%) IPG (+72%), 3.2 mA, 50 μs, 130 Hz	Lt: 1 (−2%) 2A (+9%) 2B (+4%) 2C (−1%) 3A (−58%) 3B (−22%) 3C (+22%) 4 (−17%) IPG (+65%), 4.1 mA, 50 μs, 130 Hz Rt: 1 (+2%) 2A (−4%) 2C (+1%) 3A (−96%) 3C (+7%) 4 (+6%) IPG (+84%), 3.2 mA, 50 μs, 130 Hz
13	66/M	Lt: 2A (−30%) 2B (−10%), 2C (−10%) 3A (−30%) 3B (−10%), 3C (−10%) IPG (+100%), 2.1 mA, 60 μs, 130 Hz Rt: 1 (−50%), 2A (−25%) 2C (−25%) IPG (+100%), 3.5 mA, 60 μs, 130 Hz	Lt: 1 (+10%) 2A (−29%) 2B (−29%) 2C (−29%) 3A (−4%) 3B (−5%) 3C (−4%) IPG (+86%), 2.1 mA, 60 μs, 130 Hz Rt: 1 (−42%) 2A (−55%) 2B (+11%) 2C (+9%) 3A (+11%) 3B (−1%) 3C (−1%) 4 (−1%) IPG (+69%), 3.5 mA, 60 μs, 130 Hz	Lt: 1 (+6%) 2A (−8%) 2B (−8%) 2C (−8%) 3A (−26%) 3B (−25%) 3C (−25%) 4 (+12%) IPG (+82%), 2.1 mA, 60 μs, 130 Hz Rt: 1 (−5%) 2A (−94%) 2B (+10%) 2C (−1%) 3A (+8%) 3C (+1%) IPG (+81%), 3.5 mA, 60 μs, 130 Hz

Each program is listed in the following order: contact allocation, amplitude (mA), pulse width (μs), Frequency (Hz).

### 3.2 Clinical outcomes in Part 1

In Part 1, aIGP was generated using the STN as the target region and the internal capsule as the avoid region, with the aim of reducing stimulation-induced pyramidal tract adverse effects.

After switching from the established mIGP settings to the aIGP-derived settings, 4 of the 13 patients (Cases 1, 5, 7, and 12) reported clear worsening of motor symptoms shortly after reprogramming. In these patients, UPDRS III motor assessment was performed 1 h after the change, after which the stimulation settings were returned to the original mIGP program. Among these four patients, three (Cases 5, 7, and 12) developed tremor that had not been present under the preceding mIGP settings.

The remaining nine patients did not report any marked subjective change immediately after switching to aIGP and were therefore allowed to return home. However, eight of these nine patients subsequently felt within 1 week that the previous mIGP-derived settings had been more effective and independently switched back to the original mIGP settings using the patient controller. By contrast, one patient (Case 4) did not perceive a marked change in motor symptoms, but reported improvement in speech difficulty and therefore continued using the aIGP-derived settings thereafter.

**Figure 2:**
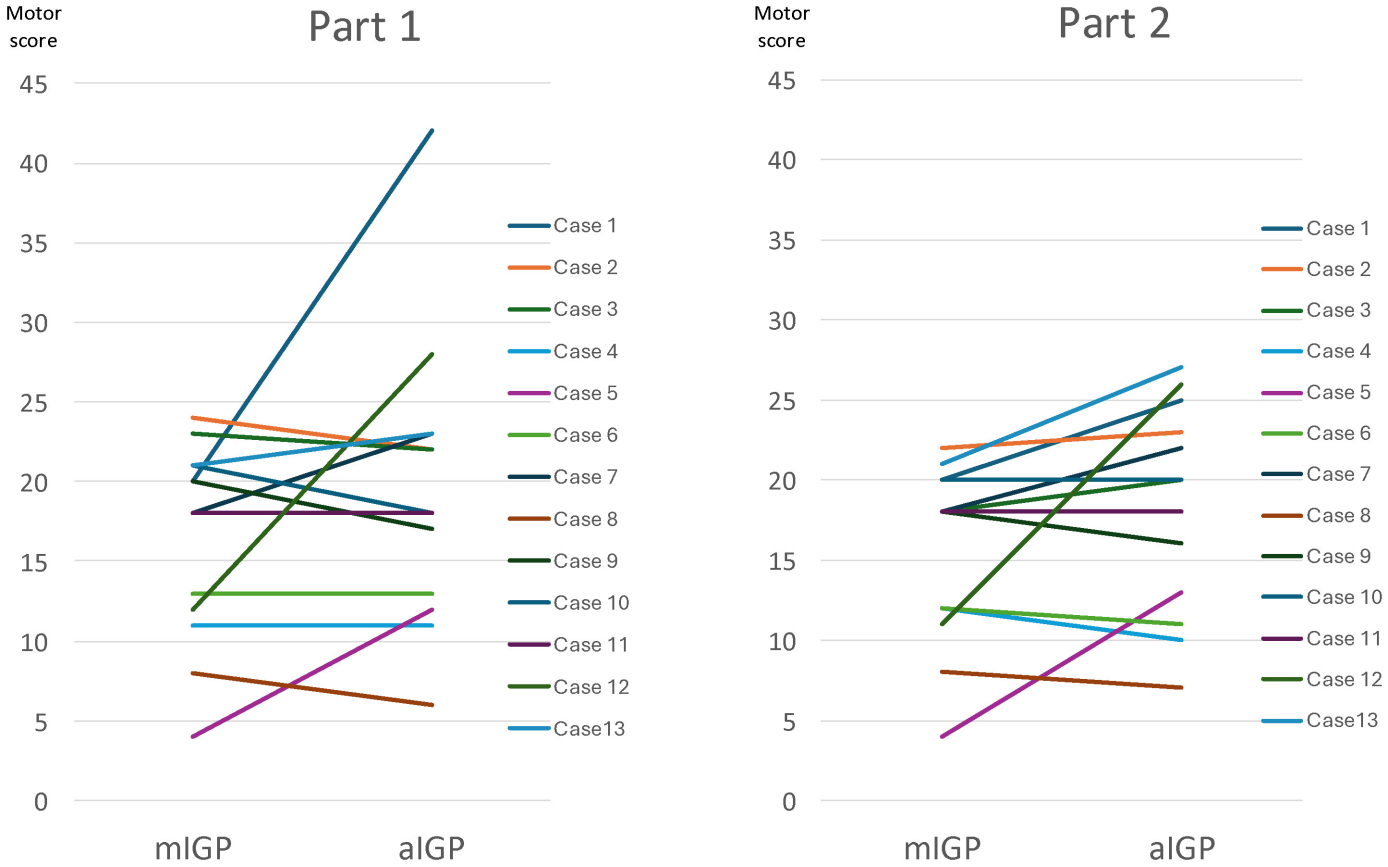
Changes in UPDRS part III motor scores 1 h after switching from mIGP to aIGP.

Changes in UPDRS III motor scores before and 1 h after switching to aIGP in Part 1 are shown in Figure 2 (left panel). The mean baseline score under mIGP was 16.4 ± 1.7, compared with 19.6 ± 2.5 at 1 h after switching to aIGP. This change did not reach statistical significance (*P* = 0.16). The planned 1-month follow-up assessment was not performed because 12 of the 13 patients had already reverted to the original mIGP settings by that time.

### 3.3 Clinical outcomes in Part 2

Part 2 was conducted on a separate occasion at least 2 months after completion of the Part 1 trial.

In Part 2, aIGP was generated using the STN as the target region and the red nucleus and substantia nigra as avoid regions, with the aim of directing the stimulation field as close as possible to the dorsolateral STN.

After switching from mIGP to the aIGP-derived settings, 3 of the 13 patients (Cases 5, 7, and 12) developed tremor shortly after reprogramming, although tremor had not been observed under the prior mIGP settings. In these patients, UPDRS III motor assessment was performed 1 hour after the switch, and the settings were then immediately returned to the original mIGP program.

The remaining 10 patients did not perceive any marked immediate change after switching to aIGP and were therefore allowed to return home. However, all 10 subsequently judged the original mIGP-derived settings to be preferable and switched back to mIGP using the patient controller within 2 weeks.

Changes in UPDRS III motor scores before and 1 h after switching to aIGP in Part 2 are shown in Figure 2 (right panel). The mean baseline score under mIGP was 15.5 ± 1.5, compared with 18.3 ± 1.8 after 1 h of aIGP. This difference was not statistically significant (*P* = 0.07). The planned 1-month follow-up assessment was not performed because all patients had returned to the original mIGP settings before that time.

## 4 Discussion

In this study, we evaluated the clinical validity of aIGP using Illumina^TM^ 3D in patients with PD who had undergone STN-DBS with a MICC directional lead system and had already achieved stable motor outcomes with mIGP using Stimview^TM^ XT. The main finding was that, although aIGP rapidly generated anatomically defined stimulation settings, these settings were not maintained in most patients. In both Part 1 and Part 2, some patients experienced immediate worsening after switching to aIGP-derived settings, whereas most of the remainder returned to their original mIGP settings within 1 to 2 weeks. These findings suggest that the current implementation of aIGP was not clinically comparable to expert-guided mIGP in terms of short-term patient acceptability and retention of the programmed settings in patients with stable chronic STN-DBS outcomes. Importantly, however, this study should not be interpreted as dismissing image-guided or anatomy-based programming itself. Rather, under the imaging and programming framework used in this study, aIGP was not clinically comparable to expert-refined mIGP in this particularly demanding cohort, in which patients had already been stabilized with mIGP.

A major explanation for the inferior performance of aIGP is that clinically optimal DBS programming cannot be determined solely by simple anatomical inclusion of the stimulation field within the STN. In the current aIGP framework, stimulation settings are generated algorithmically on the basis of predefined target and avoid regions. While this approach is attractive because of its objectivity and speed, therapeutic efficacy in actual clinical practice depends on more than anatomy alone. Effective programming requires integration of patient-specific anatomy, subtle motor responses, stimulation-induced adverse effects, and the patient's subjective experience. In particular, if the entire STN is treated as a uniform target, the algorithm may fail to provide sufficient selectivity for the dorsolateral STN, which is regarded as the most relevant motor territory (Benarroch, 2008; Guo et al., 2013; Zhang et al., 2021; Akram et al., 2017; Garcia-Garcia et al., 2016; Vitek et al., 2022). By contrast, in our mIGP strategy, stimulation was fine-tuned with explicit emphasis on the dorsolateral STN using both horizontal and vertical current steering. As long as the aIGP algorithm is designed to target the STN as a whole, it is unlikely to achieve the same degree of selective current steering toward the dorsolateral STN, and this may explain why optimization remained inferior to mIGP. In other words, anatomically plausible stimulation within the STN is not necessarily functionally optimal, because the critical issue is not simply whether the field remains within the nucleus, but whether it sufficiently engages the dorsolateral motor territory and related motor pathways.

The findings from Part 1 and Part 2 support this interpretation. In Part 1, the internal capsule was designated as the avoid region to reduce stimulation-induced pyramidal tract adverse effects. However, because the internal capsule lies lateral to the STN, this strategy may have unintentionally discouraged stimulation not only of the pyramidal tract but also of the lateral STN, thereby reducing effective engagement of the dorsolateral motor region. At the same time, one patient in Part 1 continued using the aIGP-derived settings because of improvement in dysarthria, a symptom commonly associated with pyramidal tract spread. This exceptional case suggests that, in selected patients, aIGP may indeed contribute to reduction of stimulation-related adverse effects.

In Part 2, the red nucleus and substantia nigra were selected as avoid regions in an attempt to direct stimulation toward the dorsolateral STN by constraining current spread medially and ventrally. This strategy may have shifted the stimulation field dorsally in some cases (Figure 1); however, its ability to steer stimulation sufficiently toward the lateral motor territory of the STN appeared limited. The lack of clinical improvement in Part 2 indicates that, even if the algorithm is refined to avoid adjacent non-motor structures more effectively, sufficient benefit cannot be expected unless the stimulation is explicitly optimized to maximize motor efficacy. Taken together, these findings highlight that, even in automated programming, the essential issue is not simply whether to stimulate the STN, but precisely where within the STN stimulation should be delivered. Future algorithm development should therefore move beyond simple segmentation of the STN and incorporate a more explicit representation of its motor subregion.

These results also have important clinical implications. Because aIGP can generate stimulation settings rapidly, it may be useful as an aid for initial programming or as a supportive tool in centers with limited DBS programming experience. Although not directly evaluated in the present study, aIGP may also be useful in the early postoperative period, where rapid generation of a candidate current-steering configuration could be clinically helpful. Thus, the present findings should be interpreted as showing limited performance of the current automated implementation in comparison with optimized expert programming, rather than absence of clinical utility of aIGP in all settings. However, it should be recognized that such settings are not necessarily optimal. In conventional DBS programming, clinicians generally begin with monopolar review to identify the most effective stimulation level and then adjust the stimulation field through vertical and horizontal current steering according to the patient's symptoms and complaints (Umemura et al., 2023). In contrast, algorithm-based aIGP assigns cathodic and anodic fractions to nearly all lead contacts, generating a more focused semi-monopolar configuration. Although this may reflect mathematical optimization by the algorithm, such complex settings are not typically selected in routine clinician-guided programming. This stronger focusing effect may have excessively narrowed the effective volume of tissue activated and thereby limited recruitment of sufficient motor fibers, even when the nominal target remained within the STN. Such a mechanism may help explain the new-onset tremor observed in Cases 5, 7, and 12 after switching to aIGP-derived settings. As a result, modifying these aIGP-derived configurations according to patient symptoms may be particularly challenging, especially for clinicians with limited programming experience. Thus, while aIGP may facilitate the rapid generation of candidate settings, the present results suggest that such settings may still have limited clinical acceptability and often require subsequent refinement based on patient response.

An important finding of this study was the discrepancy between the short-term quantitative assessment and the subsequent clinical acceptability of the programmed settings. In both Part 1 and Part 2, mean UPDRS III motor scores worsened after switching to aIGP-derived settings, but these changes did not reach statistical significance. However, the absence of statistical significance should not be interpreted as evidence of clinical equivalence between aIGP and mIGP. Most patients ultimately judged the original mIGP-derived settings to be preferable and reverted to them within 1 to 2 weeks, indicating that the aIGP-derived settings were not clinically acceptable for continued use in daily life. These findings suggest that a 1-h UPDRS III assessment may not be sufficiently sensitive to capture subtle but meaningful deterioration perceived by patients over time, and highlight the importance of incorporating both patient-reported outcomes and retention of the programmed settings when evaluating new DBS programming strategies. In addition, because the present cohort had already achieved stable chronic outcomes under expert-guided mIGP, the study design created a stringent benchmark condition and may have been inherently unfavorable to aIGP. The selection of this cohort may have introduced significant baseline bias in this comparative study. Furthermore, a 1-h motor assessment cannot adequately capture possible delayed effects of DBS reprogramming or non-motor changes such as mood, speech, sleep, or overall daily tolerability.

Several limitations of this study should be acknowledged. First, this was a small single-center study, and the generalizability of the findings is therefore limited. Second, outcome evaluation relied mainly on clinical observation and patient-reported symptom changes, whereas systematic quantitative assessment, including formal motor rating scales, was limited. Third, the observation period was relatively short, and long-term adaptation or delayed effects of aIGP could not be adequately evaluated. Fourth, all participants had already been stabilized with mIGP, which created a comparison condition that may have been inherently unfavorable to aIGP. Finally, the internal logic of the aIGP algorithm was not fully accessible, and therefore the detailed weighting and optimization process could not be sufficiently examined. In addition, we did not perform an independent formal manual verification of lead localization and directional orientation specifically for this study, but accepted the electrode reconstruction provided by the commercial imaging platform used for programming. Because small errors in lead localization or rotational orientation may influence estimation of the stimulation field, this should be recognized as an important methodological limitation, particularly when interpreting subtle differences between mIGP- and aIGP-derived steering patterns. Future studies should therefore incorporate independent validation of lead localization and directional orientation, ideally using validated manual approaches (Fayed et al., 2023), together with quantitative spatial comparison of stimulation fields and, where feasible, tract-informed analysis of engagement of the dorsolateral STN and related motor pathways.

Despite these limitations, the present study provides clinically relevant insight into the current strengths and limitations of automated programming in STN-DBS. Our findings suggest that, although aIGP rapidly generated anatomically plausible current-steering configurations, most patients did not maintain these settings in daily life, indicating limited clinical acceptability compared with expert-guided mIGP. At the same time, these results should not be interpreted as rejecting image-guided or anatomy-based programming itself, but rather as indicating that the current automated implementation was insufficient to match expert-refined manual image-guided programming under the conditions tested here. Further refinement of automated algorithms, including more selective emphasis on the dorsolateral motor territory of the STN, better incorporation of functional motor circuitry, and rigorous validation of anatomical input data such as lead localization and orientation, will be required before aIGP can be adopted as a reliable alternative to expert-based programming.

## 5 Conclusions

In conclusion, the present study showed that aIGP can rapidly generate anatomically reasonable current-steering configurations in STN-DBS, but its current clinical performance was not clinically comparable to expert-guided mIGP in this cohort. These findings suggest that successful DBS programming depends not only on anatomical visualization of the STN, but also on selective engagement of the most effective motor subregion, particularly the dorsolateral STN, together with clinically informed fine-tuning.

## Data Availability

The original contributions presented in the study are included in the article/supplementary material, further inquiries can be directed to the corresponding author.

## References

[B1] Akram H. Sotiropoulos S. N. Jbabdi S. Georgiev D. Mahlknecht P. Hyam J. . (2017). Subthalamic deep brain stimulation sweet spots and hyperdirect cortical connectivity in Parkinson's disease. Neuroimage. 158, 332–345. doi: 10.1016/j.neuroimage.2017.07.012 28711737 PMC6581538

[B2] Aldred J. L. Zesiewicz T. Okun M. S. Ramirez-Zamora A. Vaou O. E. Verhagen Metman L. . (2025). Sustained therapeutic benefits using image-guided programming at activation of deep brain stimulation for Parkinson's disease. Mov. Disord. Clin. Pract. 12, 1821–1830. doi: 10.1002/mdc3.70154 40474569 PMC12625106

[B3] Benarroch E. E. (2008). Subthalamic nucleus and its connections: anatomic substrate for the network effects of deep brain stimulation. Neurology. 70, 1991–1995. doi: 10.1212/01.wnl.0000313022.39329.65 18490619

[B4] Dembek T. A. Reker P. Visser-Vandewalle V. Wirths J. Treuer H. Klehr M. . (2017). Directional DBS increases side-effect thresholds-a prospective, double-blind trial. Mov. Disord. 32, 1380–1388. doi: 10.1002/mds.27093 28843009

[B5] Fayed I. Syed M. Gingold E. Alizadeh M. Sharan A. Wu C. (2023). A novel and simple method using computed tomography streak artifact to determine the orientation of directional deep brain stimulation leads. Neurosurgery. 93, 1036–1045. doi: 10.1227/neu.0000000000002536 37227135

[B6] Garcia-Garcia D. Guridi J. Toledo J. B. Alegre M. Obeso J. A. Rodríguez-Oroz M. C. (2016). Stimulation sites in the subthalamic nucleus and clinical improvement in Parkinson's disease: a new approach for active contact localization. J. Neurosurg. 125, 1068–1079. doi: 10.3171/2015.9.JNS15868 26848922

[B7] Guo S. Zhuang P. Hallett M. Zheng Z. Zhang Y. Li J. . (2013). Subthalamic deep brain stimulation for Parkinson's disease: correlation between locations of oscillatory activity and optimal site of stimulation. Parkinsonism Relat. Disord. 19, 109–114. doi: 10.1016/j.parkreldis.2012.08.005 22981262

[B8] Lange F. Steigerwald F. Malzacher T. Brandt G. A. Odorfer T. M. Roothans J. . (2021). Reduced programming time and strong symptom control even in chronic course through imaging-based DBS programming. Front. Neurol. 12:785529. doi: 10.3389/fneur.2021.785529 34819915 PMC8606823

[B9] Maçaneiro M. T. Azevedo A. C. Poerner B. M. da Silva M. D. Koerbel A. (2024). Directional deep brain stimulation in the management of Parkinson's disease: efficacy and constraints-an analytical appraisal. Neurosurg. Rev. 47:43. doi: 10.1007/s10143-023-02268-x 38216697

[B10] Maghzi H. Kim C. Worthge S. Malatt C. Tagliati M. (2025). Traditional deep brain stimulation programming versus automated image-guided algorithm in patients with Parkinson's disease [abstract]. Mov. Disord. 40(Suppl. 1). Available online at: https://www.mdsabstracts.org/abstract/traditional-deep-brain-stimulation-programming-versus-automated-image-guided-algorithm-in-patients-with-parkinsons-disease/ (Accessed March 27, 2026).

[B11] Malekmohammadi M. Mustakos R. Sheth S. Pouratian N. McIntyre C. C. Bijanki K. R. . (2022). Automated optimization of deep brain stimulation parameters for modulating neuroimaging-based targets. J. Neural Eng. 19:10.1088/1741-2552/ac7e6c. doi: 10.1101/2022.05.23.22275220 PMC11090244 35790135

[B12] Merola A. Romagnolo A. Krishna V. Pallavaram S. Carcieri S. Goetz S. . (2020). Current directions in deep brain stimulation for Parkinson's disease-directing current to maximize clinical benefit. Neurol. Ther. 9, 25–41. doi: 10.1007/s40120-020-00181-9 32157562 PMC7229063

[B13] Pavese N. Tai Y. F. Yousif N. Nandi D. Bain P. G. (2020). Traditional trial and error versus neuroanatomic 3-dimensional image software-assisted deep brain stimulation programming in patients with Parkinson disease. World Neurosurg. 134, e98–e102. doi: 10.1016/j.wneu.2019.09.106 31568905

[B14] Rolland A. S. Touzet G. Carriere N. Mutez E. Kreisler A. Simonin C. . (2024). The use of image guided programming to improve deep brain stimulation workflows with directional leads in Parkinson's disease. J. Parkinsons Dis. 14, 111–119. doi: 10.3233/JPD-225126 38189764 PMC10836544

[B15] Schnitzler A. Mir P. Brodsky M. A. Verhagen L. Groppa S. Alvarez R. . (2022). Directional deep brain stimulation for Parkinson's disease: results of an international crossover study with randomized, double-blind primary endpoint. Neuromodulation. 25, 817–828. doi: 10.1111/ner.13407 34047410

[B16] Torres V. Del Giudice K. Roldán P. Rumià J. Muñoz E. Cámara A. . (2024). Image-guided programming deep brain stimulation improves clinical outcomes in patients with Parkinson's disease. NPJ Parkinsons Dis. 10:29. doi: 10.1038/s41531-024-00639-9 38280901 PMC10821897

[B17] Umemura A. Mizuno H. Maki M. Masago A. (2025). Image-guided optimization of current steering in STN-DBS for Parkinson's disease. Front. Neurol. 16:1618480. doi: 10.3389/fneur.2025.1618480 40672446 PMC12263581

[B18] Umemura A. Oka Y. Yamada K. Oyama G. Shimo Y. Hattori N. (2013). Validity of single tract microelectrode recording in subthalamic nucleus stimulation. Neurol. Med. Chir. 53, 821–827. doi: 10.2176/nmc.oa2012-0412 PMC4508719 24140767

[B19] Umemura A. Oyama G. Iwamuro H. Shimo Y. Hatano T. Kamo H. . (2023). Application of current steering with MICC directional lead in STN-DBS for Parkinson's disease. Deep Brain Stimul. 1, 20–25. doi: 10.1016/j.jdbs.2023.03.002

[B20] Vilkhu G. Goas C. Miller J. A. Kelly S. M. McDonald K. J. Tsai A. J. . (2023). Clinician vs. imaging-based subthalamic nucleus deep brain stimulation programming. Parkinsonism Relat. Disord. 106:105241. doi: 10.1016/j.parkreldis.2022.105241 36525899

[B21] Vitek J. L. Patriat R. Ingham L. Reich M. M. Volkmann J. Harel N. (2022). Lead location as a determinant of motor benefit in subthalamic nucleus deep brain stimulation for Parkinson's disease. Front. Neurosci. 16:1010253. doi: 10.3389/fnins.2022.1010253 36267235 PMC9577320

[B22] Waldthaler J. Bopp M. Kühn N. Bacara B. Keuler M. Gjorgjevski M. . (2021). Imaging-based programming of subthalamic nucleus deep brain stimulation in Parkinson's disease. Brain Stimul. 14, 1109–1117. doi: 10.1016/j.brs.2021.07.064 34352356

[B23] Zhang F. Wang F. Li W. Wang N. Han C. Fan S. . (2021). Relationship between electrode position of deep brain stimulation and motor symptoms of Parkinson's disease. BMC Neurol. 21:122. doi: 10.1186/s12883-021-02148-1 33731033 PMC7972210

